# Association of State Funding for Comprehensive Reproductive Health Care With Use of Contraception Among Latina Patients and Non-Latina Patients in Oregon

**DOI:** 10.1001/jamahealthforum.2023.2144

**Published:** 2023-07-28

**Authors:** Megan A. Cohen, Emily R. Boniface, Megan Skye, Rachel Linz, Nisreen Pedhiwala, Maria I. Rodriguez

**Affiliations:** 1 Department of Obstetrics and Gynecology, Oregon Health & Science University, Portland; 2 Department of Gynecology and Obstetrics, Emory University, Atlanta, Georgia; 3 Reproductive Health Program, Oregon Health Authority, Portland; 4 Center for Health Systems Effectiveness, Oregon Health & Science University, Portland

## Abstract

**Importance:**

There are well-documented racial and ethnic disparities in effective contraceptive use due to multiple factors. A key contributor may be differential insurance coverage and patient costs. The Oregon Reproductive Health Equity Act (RHEA), enacted in 2017, ensures full coverage of reproductive health care without cost sharing for all Oregonians with low income.

**Objective:**

To assess whether removing financial barriers to accessing contraceptive care is associated with an improvement in use of moderately and highly effective contraceptive methods among Latina women compared with non-Latina women.

**Design, Setting, and Participants:**

This cohort study included women aged 12 to 51 years who sought contraceptive care in the Oregon Health Authority Reproductive Health Program from April 2016 to March 2020. Patients using permanent contraception, those missing data on ethnicity, and non-Oregon residents were excluded. Data analysis was performed in January 2021.

**Exposures:**

Contraceptive care in the Reproductive Health Program after RHEA implementation.

**Main Outcomes and Measures:**

The main outcome was prevalence of use of moderately effective methods (contraceptive pills, patch, ring, or injection) or highly effective methods (long-acting reversible contraceptives) at the time of the contraceptive visit. Difference-in-differences analysis was used to compare moderately and highly effective contraception use between Latina and non-Latina patients. Secondary outcomes were proportions of adoption or continuation of moderately and highly effective methods, prevalence of highly effective methods, and adoption or continuation of highly effective methods. The unit of analysis was at the clinic visit level.

**Results:**

The study sample consisted of 295 604 evaluable clinic visits, in which 28.4% of individuals identified as Latina and 71.6% of individuals identified as non-Latina. The mean (SD) age was 25.5 (8.1) years. With difference-in-differences analysis, there was a significant adjusted 1.9 percentage point (95% CI, 0.2-3.6 percentage points) increase in prevalence of moderately or highly effective contraceptive methods for Latina patients compared with non-Latina patients. There were no significant differences for other outcomes of interest.

**Conclusions:**

This cohort study found that in Oregon, legislation removing financial barriers to accessing contraceptive care was associated with significant mitigation in disparate moderately and highly effective contraceptive method prevalence for Latina patients compared with non-Latina patients. The findings suggest that state funds successfully supplanted federal funds to enable continued robust comprehensive family planning services after withdrawal from Title X.

## Key Points

**Question:**

Is comprehensive reproductive health care that is covered for all Oregonians with low income regardless of citizenship status associated with an improvement in use of moderately and highly effective contraceptive methods among Latina women?

**Findings:**

In this cohort study of 295 604 clinic visits for contraception in Oregon, implementation of legislation ensuring comprehensive contraception coverage without cost sharing was associated with an increase in moderately and highly effective contraceptive use among Latina patients compared with non-Latina patients.

**Meaning:**

In this study, when contraceptive costs were fully covered for all individuals with low income, more Latina women than non-Latina women used moderately and highly effective contraceptive methods.

## Teaser

This cohort study investigates whether state funding for comprehensive reproductive health care is associated with an improvement in use of moderately and highly effective contraceptive methods among Latina women.

## Introduction

Contraception is an essential tool to promote reproductive autonomy,^1,2^ but significant racial and ethnic disparities in contraceptive use exist in the US.^3,4,5,6,7^ In 2016, 59.9% of US Black women and 58.1% of Latina women were using moderately or highly effective methods of contraception compared with 62.9% of non-Latina White women.^8^ Multiple factors at the individual, community, and health system levels are associated with differences in contraceptive use, including racism within the health system that perpetuates disparities.^9,10,11^ Evidence shows that cost and other financial barriers (eg, lack of insurance, child care, time away from work, and transportation challenges) are associated with nonuse of contraception,^12,13^ but it is unclear to what extent differences in coverage of contraceptive costs are associated with disparities in contraceptive use compared with patient preferences or other factors. Historically marginalized populations, including Latina women and non-Latina Black women, are significantly more likely to be uninsured or enrolled in Medicaid compared with non-Latina White women.^14^

Nationally, Medicaid expansion under the Patient Protection and Affordable Care Act (ACA) improved access to reproductive health care and contraception markedly.^15^ However, enrollment in Medicaid requires not just evidence of having low income but also proof of identity and US citizenship or a certain eligible immigration status, such as lawful permanent residency or refugee status, documentation that many individuals in need of publicly funded family planning may not have or qualify for. Due to coverage gaps even after ACA implementation, many individuals relied on Title X funding to obtain contraceptive services because the federal program provides low-cost contraception without requiring proof of citizenship, immigration status, residency, or income.^16,17^ This access was threatened after reinstitution of a restrictive federal policy led to defunding of approximately 25% of Title X clinics and led 6 states, including Oregon, to withdraw completely from the Title X program in August 2019.^18^ Oregon replaced Title X funding with state funding to maintain services, allocated from funds established by a novel policy enacted in 2017, the Oregon Reproductive Health Equity Act (RHEA).

RHEA, which reached full implementation in April 2018, provides a legal framework for ensuring that all state residents who meet financial requirements (<250% of the federal poverty level)^19^ are entitled to their choice of no-cost contraception. RHEA includes 2 main provisions to reduce financial barriers to accessing contraception: (1) it requires commercial insurance plans to cover contraception with no patient cost sharing, and (2) it provides funding to cover reproductive health services for individuals who would otherwise be eligible for Medicaid coverage if not for their immigration status. This funding helps support Oregon’s Reproductive Health (RH) Program, a statewide network of 150 clinics, which are access points for evidence-based care that provide a full range of contraceptive methods.^20^

RHEA helped expand contraceptive access compared with Title X in several ways. Title X funds had been distributed as grant funds prior to RHEA implementation using a funding formula based on the number of patients served in previous years, so it could take more than a year for changes in clinic needs to be reflected in the amount of Title X funds available for that specific clinic. With the implementation of RHEA, the Oregon RH Program switched to a fee-for-service model that was more responsive to changes in patient volume and contraceptive method choices. As an example, due to limited funding and specific provisions within the Title X program, certain Oregon RH Program clinics were unable to meet monthly patient demand for long-acting reversible contraceptives prior to RHEA implementation. Additionally, the RHEA funding made available to the RH Program was almost twice as much as the Title X grant award had been.

The purpose of this study was to examine the association of RHEA implementation with disparities in use of moderately or highly effective contraception among Latina women compared with non-Latina women. We focused on Latina women because they comprise the largest racial and ethnic minority group in Oregon; they are most likely to rely on publicly funded clinics for contraceptive care^17^ and are the demographic group in Oregon most likely to benefit from RHEA contraceptive funding. We used data from the Oregon Health Authority’s RH Program to characterize differences in moderately and highly effective contraceptive use by ethnicity. We hypothesized that Latina women would experience a greater increase in use of moderately and highly effective contraception than non-Latina women in association with RHEA implementation.

## Methods

We conducted a historical cohort study of clinic visits for women aged 12 to 51 years seeking contraceptive services in Oregon’s publicly funded family planning program. The study period was from April 2016 to March 2020 to allow for 2 years of data before and after full implementation of expanded contraceptive coverage under RHEA in April 2018. The unit of analysis was at the clinic visit level. We performed our analysis in January 2021 after receiving completed data from the Oregon RH Program. The study was approved by the institutional review board at the Oregon Health Authority and deemed exempt by the institutional review board at Oregon Health & Science University owing to the large sample size with minimal location identifiers. Requirement for informed consent of participants was therefore waived. We followed the Strengthening the Reporting of Observational Studies in Epidemiology (STROBE) reporting guideline.^21^

The study population focused on individuals at risk of pregnancy. We excluded visits for patients who identified as male, female patients using permanent contraception, and female patients younger than 12 years or older than 51 years. We excluded individuals who had zip codes that were not located within Oregon or were invalid as they would not be eligible for RHEA coverage and patients who had missing ethnicity data (eFigure 1 in Supplement 1).

We obtained demographic and clinical variables from the program’s clinic visit record (CVR), a standardized form that is required for each visit. The CVR contains a wide array of information on patient demographics, insurance status, payer source, clinical information such as services rendered during the visit, and clinic-level variables. It includes information on the contraceptive method being used at the start and end of the clinic visit, including the full array of US Food and Drug Administration–approved contraceptive methods and behavioral methods such as withdrawal. We additionally used RH Program enrollment forms to cross-reference patient demographic information and zip code data.

### Variables

Our primary independent variable was Latina or non-Latina ethnicity given inconsistent data on immigration status, particularly prior to RHEA implementation. We therefore focused our analysis on Latina patients compared with non-Latina patients, as Latino individuals are the largest ethnic minority in Oregon and prior studies have shown that more than 80% to 90% of individuals benefiting from similar state emergency Medicaid coverage identified as Hispanic or Latina.^22,23^ Ethnicity is a self-reported variable on the CVR, which we cross-referenced with RH Program enrollment forms. We classified an individual as Latina if they reported “Hispanic or Latino” on the CVR at any visit or on the RH Program enrollment form. We collapsed race and ethnicity into a single variable to identify Latina individuals in the program. We did not otherwise exclude individuals of any specific race from the non-Latina comparison group given expected low proportions of racial and ethnic minority individuals and a desire to not introduce bias into the sample. The immigrant population (who would be most affected by the RHEA legislation) within Oregon is predominantly Latino, so the non-Latino population is exceedingly small. We anticipated low proportions of individuals who were from other racial or ethnic minority groups. Additionally, the difference-in-differences (DID) method requires 2 separate groups for analysis. Therefore, we used Latina and non-Latina ethnicity as the binary comparison. Race categories were Alaska Native, American Indian, Asian, Black, Native Hawaiian or Pacific Islander, White, other, or unknown or not reported. Other is a specific category available on the CVR, so no additional level of detail is available.

Our primary outcome of interest was prevalence of use of a moderately or highly effective contraceptive method. We measured prevalence as the method listed at the start of the clinic visit. Secondary outcomes included use of a moderately or highly effective form of contraception at the end of the visit (adoption or continuation of the method), prevalence of a highly effective method at the start of the visit, and adoption or continuation of a highly effective method. Moderately effective methods included combined hormonal contraceptive methods (combined pills, patch, and ring), progestin-only pills, and injectable contraception. Highly effective reversible methods included intrauterine devices and hormonal implants. For descriptive analyses, other methods were classified as barrier methods (condom, diaphragm, contraceptive sponge, and spermicide), withdrawal or natural methods (natural family planning, fertility awareness, and lactational amenorrhea), no method (none, abstinence, or unknown method), or other method.

We chose method prevalence as our primary outcome to better evaluate effects of the RHEA policy itself instead of method adoption and continuation, which we thought more reflected the quality of the RH Program. We did not use switching from a less effective to highly effective contraceptive method as our primary outcome as it is not a person-centered measure and may result from reproductive coercion. However, we included highly effective method prevalence and adoption or continuation as secondary outcome measures since long-acting reversible contraceptives are more costly than short-acting methods, and therefore, their use can be indicative of adequate cost coverage for patients.

We created a time variable before and after RHEA implementation, using April 2018 as the time of full RHEA implementation. We then modeled this time variable in an interaction with ethnicity as described below.

We abstracted additional demographic and clinical data from the CVR, including patient sex, patient age, self-reported zip code, self-reported monthly income and household size, need for an interpreter, payer source (private insurance or payment of full fee out of pocket, Medicaid, RHEA, and Title X), and insurance type (public, private, uninsured, or unknown). We did not have information on patient self-identified gender. We modeled patient age continuously. We calculated household federal poverty level category (<138%, 139%-249%, and ≥250%) using monthly income and household size.^24^ We coded urban or rural status binarily as metropolitan or nonmetropolitan by classifying zip codes according to Rural-Urban Commuting Area codes.^25^

### Statistical Analysis

We used descriptive analyses and data visualizations to assess trends in contraceptive use, initiation, and continuation over time. We examined trends in the payer (eg, Medicaid, Title X) for contraceptive visits across the study period: before full RHEA implementation (prior to April 2018), after full RHEA implementation (after April 2018), and after Oregon’s withdrawal from Title X (August 2019). We characterized the population overall and stratified by race and ethnicity.

To compare changes in contraceptive outcomes associated with the policy change, we used DID design. Difference-in-differences analyses relied on the assumption that prepolicy trends in contraceptive use between Latina individuals and non-Latina individuals were parallel. We conducted tests of parallel trends for all study outcomes and found that the parallel trends assumption held for all outcomes prior to the policy change. We selected covariates for models based on clinical or reported associations with our outcomes. In models for all study outcomes, we adjusted for patient age, metropolitan status, and federal poverty level category.^26,27,28^ We clustered SEs at the clinic level. Parity is associated with incident highly effective contraception use,^26^ but we were unable to measure this covariate as it is not captured on the CVR. For all of our DID models, we conducted 2-sided tests with α = .05. We additionally performed a sensitivity analysis excluding the first quarter of 2020 since this coincided with the beginning of the COVID-19 pandemic, which affected health care utilization. Analyses were performed in R, version 4.1.1 (R Foundation for Statistical Computing).

## Results

The initial cohort included 328 982 visits; 9977 were excluded for patients who identified as male; 4300, for female patients using permanent contraception; 1514, for female patients younger than 12 years or older than 51 years; 5063, for individuals who had zip codes that were not located within Oregon or were invalid; and 12 524 for patients who had missing ethnicity data. The analytic cohort consisted of 295 604 clinic visits for women aged 12 to 51 years presenting for contraceptive services (eFigure 1 in Supplement 1). A total of 84 013 (28.4%) were for individuals who identified as Latina, and 211 591 clinic visits (71.6%) were for individuals who identified as non-Latina. The mean (SD) age of Latina patients was 27.8 (8.9) years compared with 24.6 (7.6) years for non-Latina patients. For the entire cohort, the mean (SD) age was 25.5 (8.1) years. The racial breakdown differed between the ethnicity groups (Table 1). Among Latina patients, 31 (0.04%) identified as Alaska Native, 1283 (1.5%) as American Indian, 360 (0.4%) as Asian, 844 (1.0%) as Black, 1175 (1.4%) as Native Hawaiian or Pacific Islander, 49 399 (58.8%) as White, and 12 727 (15.1%) as other; 18 224 (21.7%) reported unknown race or did not report race. Of individuals with non-Latina ethnicity, 112 (0.05%) identified as Alaska Native, 2041 (1.0%) as American Indian, 10 977 (5.2%) as Asian, 9493 (4.5%) as Black, 1427 (0.7%) as Native Hawaiian or Pacific Islander, 174 280 (82.4%) as White, and 2626 (1.2%) as other; for 10 635 (5.0%), race was unknown or not reported.

**Table 1.** Demographic Characteristics of Patients Seeking Contraception in Oregon by Latina or Non-Latina Ethnicity, 2016-2020

Patient characteristics per visit	Patients, No. (%)		
	Latina (n = 84 013)	Non-Latina (n = 211 591)	Overall (N = 295 604)
Age, mean (SD), y	27.8 (8.9)	24.6 (7.6)	25.5 (8.1)
Race and ethnicity			
Alaska Native	31 (0.04)	112 (0.05)	143 (0.05)
American Indian	1283 (1.5)	2041 (1.0)	3324 (1.1)
Asian	360 (0.4)	10 977 (5.2)	11 337 (3.8)
Black	844 (1.0)	9493 (4.5)	10 337 (3.5)
Native Hawaiian or Pacific Islander	1175 (1.4)	1427 (0.7)	2602 (0.9)
White	49 399 (58.8)	174 280 (82.4)	223 649 (75.7)
Other^a^	12 727 (15.1)	2626 (1.2)	15 353 (5.2)
Unknown or not reported	18 224 (21.7)	10 635 (5.0)	28 859 (9.8)
Federal poverty level, %			
<138	68 257 (81.2)	159 946 (75.6)	228 203 (77.2)
138-249	13 098 (15.6)	42 821 (20.2)	55 919 (18.9)
≥250	2494 (3.0)	8222 (3.9)	10 716 (3.6)
Insurance type			
Private	9061 (10.8)	54 119 (25.6)	63 180 (21.4)
Public	20 199 (24.0)	66 440 (31.4)	86 639 (29.3)
Uninsured	53 376 (63.5)	76 881 (36.3)	130 257 (44.1)
Unknown	1377 (1.6)	14 151 (6.7)	15 528 (5.3)
Need interpreter			
No	49 315 (58.7)	205 337 (97.0)	254 652 (86.1)
Yes	34 698 (41.3)	6254 (3.0)	40 952 (13.9)
Zip code			
Metropolitan	62 007 (73.8)	155 480 (73.5)	217 487 (73.6)
Nonmetropolitan	21 939 (26.1)	55 842 (26.4)	77 781 (26.3)
Source of payment			
Private insurance or full fee	6435 (7.7)	20 249 (9.6)	26 684 (9.0)
Medicaid	40 740 (48.5)	175 588 (83.0)	216 328 (73.2)
RHEA	16 191 (19.3)	3923 (1.9)	20 114 (6.8)
Title X	20 647 (24.6)	11 831 (5.6)	32 478 (11.0)

Abbreviation: RHEA, Reproductive Health Equity Act.

^a^ The “other” category was taken from the clinic visit record, in which it is its own specific category that can be marked; thus, there is no further level of detail available.

The majority of clinic visits were made by patients who were at less than 138% of the federal poverty level (68 257 [81.2%] for Latina patient visits and 159 946 [75.6%] for non-Latina patient visits) and lived in metropolitan areas (62 007 [73.8%] for Latina patient visits and 155 480 [73.5%] for non-Latina patient visits) (Table 1). Only 76 881 non-Latina patient visits (36.3%) were for uninsured patients compared with 53 376 visits (63.5%) for Latina patients. Medicaid served as a source of payment for a greater proportion of non-Latina patient visits (175 588 [83.0%]) compared with Latina patient visits (40 740 [48.5%]). Conversely, RHEA funds served as a source of payment for fewer non-Latina patient visits compared with Latina patient visits (3923 [1.9%] vs 16 191 [19.3%]), as did Title X (11 831 [5.6%] vs 20 647 [24.6%]) (Table 1).

**Figure 1.**
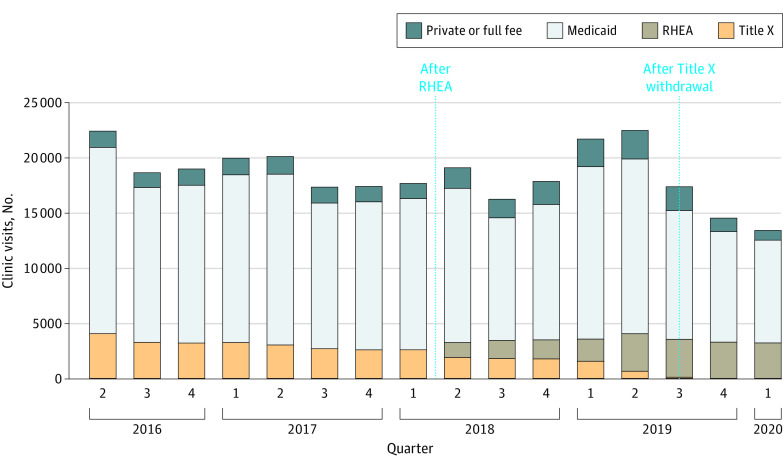
Number of Contraceptive Visits by Source of Payer per Quarter in Oregon, 2016-2020 The cohort included 295 604 clinic visits. RHEA indicates Reproductive Health Equity Act.

Overall clinic visits in Oregon’s publicly funded RH Program remained relatively stable before and after RHEA implementation (Figure 1). Medicaid funds paid for the largest proportion of clinic visits throughout the entire study period. Prior to RHEA implementation, Title X funds represented the second largest payer source (Figure 1). RHEA funding began supplanting Title X funding after RHEA implementation in April 2018 and completely replaced Title X as a payer source after Oregon’s Title X withdrawal. Although state funds fully replaced Title X expenditures, a small decline in overall clinic visits was observed following Title X withdrawal (Figure 1). The last quarter of the period included the start of the COVID-19 pandemic during March 2020, when overall access to health care declined.

During the majority of all visits both before and after policy implementation, patients adopted or continued a moderately or highly effective contraceptive method (Table 2). Latina patients had a greater prevalence of highly effective method use throughout the study period compared with non-Latina patients. Prevalence of highly effective method use increased for both Latina and non-Latina patient visits before and after policy implementation (Latina patients: from 8343 [20.4%] to 10 299 [23.8%]; non-Latina patients: from 18 875 [16.9%] to 19 448 [19.5%]). The full, unadjusted contraceptive method mix for non-Latina patients and Latina patients stratified by policy period is given in Table 2.

**Table 2.** Previsit and Postvisit Contraception for Patients Seeking Contraception in Oregon by RHEA Time Point, 2016-2020

	Clinic visits, No. (%)			
	Latina		Non-Latina	
	Before RHEA (n = 40 808)	After RHEA (n = 43 205)	Before RHEA (n = 111 924)	After RHEA (n = 99 667)
Outcome				
Prevalence of moderately and highly effective methods^a^^,^^b^	23 646 (57.9)	25 649 (59.4)	68 413 (61.1)	60 571 (60.8)
Adoption and continuation of moderately and highly effective methods^a^^,^^b^	31 840 (78.0)	33 128 (76.7)	93 101 (83.2)	80 378 (80.6)
Prevalence of highly effective methods^a^	8343 (20.4)	10 299 (23.8)	18 875 (16.9)	19 448 (19.5)
Adoption and continuation of highly effective methods^a^	9983 (24.5)	11 410 (26.4)	24 600 (22.0)	23 645 (23.7)
Prevalent contraceptive method				
IUD or IUS	4578 (11.2)	5249 (12.1)	10 824 (9.7)	10 807 (10.8)
Implant	3765 (9.2)	5050 (11.7)	8051 (7.2)	8641 (8.7)
Injectable	7138 (17.5)	7597 (17.6)	18 407 (16.4)	15 995 (16.0)
Other moderately effective methods^c^	8159 (20.0)	7748 (17.9)	31 107 (27.8)	25 101 (25.2)
Barrier methods^d^	6819 (16.7)	6391 (14.8)	19 875 (17.8)	15 269 (15.3)
Withdrawal or natural methods^e^	935 (2.3)	1091 (2.5)	2353 (2.1)	2813 (2.8)
No method^f^	9273 (22.7)	9883 (22.9)	20 831 (18.6)	20 727 (20.8)
Other method	141 (0.3)	196 (0.5)	476 (0.4)	314 (0.3)

Abbreviations: IUD, intrauterine device; IUS, intrauterine system; RHEA, Reproductive Health Equity Act.

^a^ Highly effective methods included IUD or IUS and implant.

^b^ Moderately effective methods included injectable contraceptive, oral contraceptive pills, contraceptive patch, and contraceptive ring.

^c^ Other moderately effective methods included oral contraceptive pills, contraceptive patch, and contraceptive ring.

^d^ Barrier methods included male condom, female condom, diaphragm, contraceptive sponge, and spermicide.

^e^ Withdrawal or natural methods included withdrawal, lactation amenorrhea method, natural family planning, and fertility awareness method.

^f^ No method included abstinence, no method, and unknown method.

**Figure 2.**
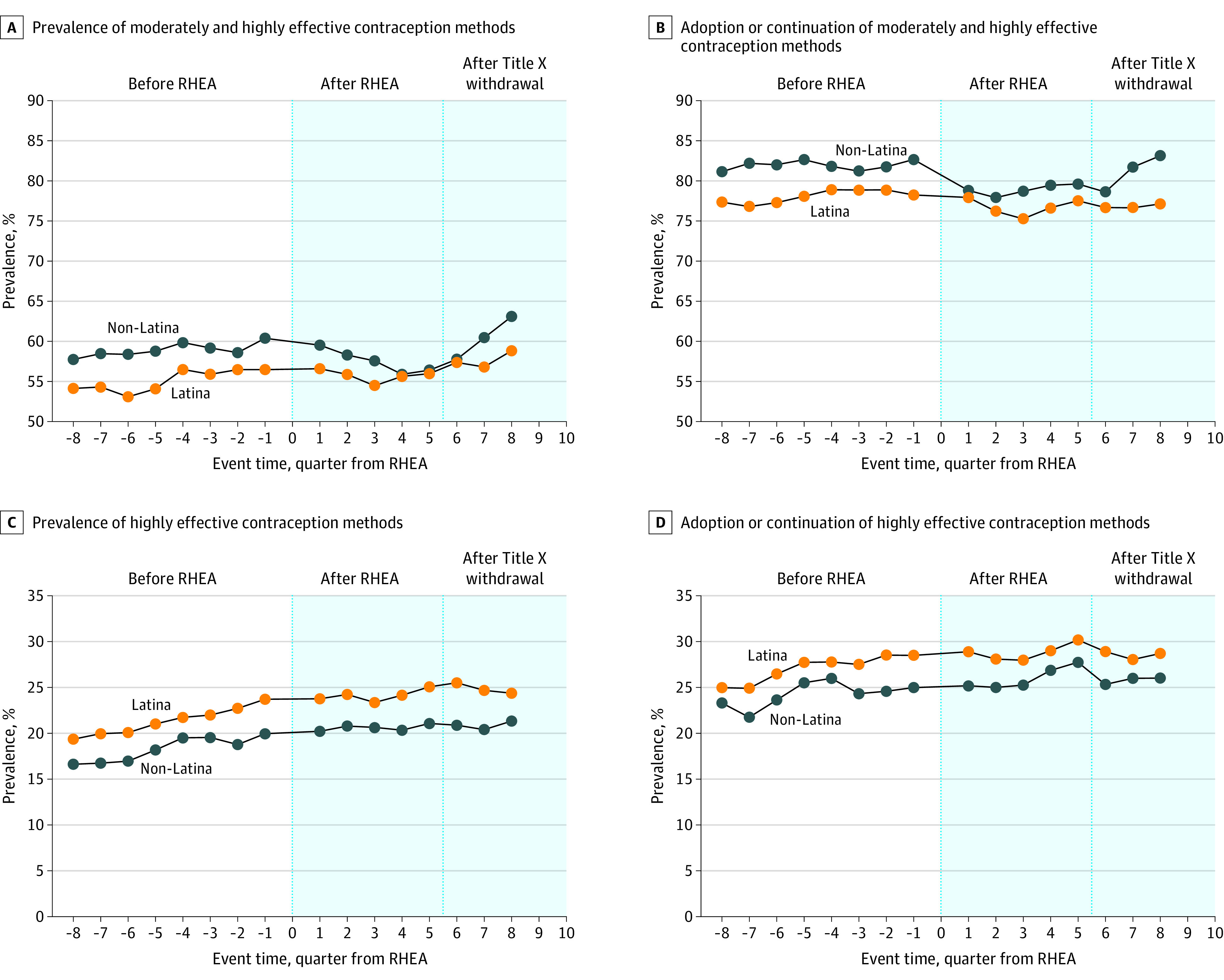
Adjusted Trend Estimates for Latina Patients Compared With Non-Latina Patients by Quarter, 2016-2020 The shaded area represents the time after implementation of the Reproductive Health Equity Act (RHEA).

Table 3 shows the adjusted DID estimates for changes in contraceptive outcomes. Moderately and highly effective contraceptive method prevalence for non-Latina patient visits remained consistent before and after RHEA implementation (68 413 [61.1%] and 60 571 [60.8%], respectively) while slightly increasing for Latina patient visits (23 646 [57.9%] and 25 649 [59.4%], respectively). In our adjusted DID model, we estimated that this corresponded to an increase of 1.9 percentage points (95% CI, 0.2-3.6 percentage points) for prevalence of moderately or highly effective contraception use for Latina patients compared with non-Latina patients (Table 3 and Figure 2A). We did not observe a significant difference in other outcomes of interest, including adoption or continuation of a moderately or highly effective method during the contraceptive visit, use of a highly effective method, and adoption or continuation of a highly effective method (Table 3 and Figure 2B-D). Graphic depictions of the adjusted trend estimates over time for each outcome are shown for Latina and non-Latina patients in Figure 2 (unadjusted trend estimates are shown in eFigure 2 in Supplement 1). There appeared to be a slight decrease in the prevalence of moderate and effective method use for non-Latina patients in the fourth through sixth quarters after RHEA implementation, without a corresponding change for Latina patients. Secular trends for the remainder of the outcomes and throughout the rest of the study period did not differ.

**Table 3.** Changes in Contraceptive Outcomes for Latina Women Compared With Non-Latina Women in Oregon Following RHEA Implementation, 2016-2020

Outcome	Clinic visits for Latina women, No. (%)		Clinic visits for non-Latina women, No. (%)		Adjusted DID estimate, percentage points (95% CI)^a^
	Before RHEA (n = 40 808)	After RHEA (n = 43 205)	Before RHEA (n = 111 924)	After RHEA (n = 99 667)	
Prevalence of moderately and highly effective methods^b^^,^^c^	23 646 (57.9)	25 649 (59.4)	68 413 (61.1)	60 571 (60.8)	1.9 (0.2 to 3.6)
Adoption and continuation of moderately and highly effective methods^b^^,^^c^	31 840 (78.0)	33 128 (76.7)	93 101 (83.2)	80 378 (80.6)	1.1 (−0.6 to 2.8)
Prevalence of highly effective methods^b^	8343 (20.4)	10 299 (23.8)	18 875 (16.9)	19 448 (19.5)	0.6 (−0.4 to 1.7)
Adoption and continuation of highly effective methods^b^	9983 (24.5)	11 410 (26.4)	24 600 (22.0)	23 645 (23.7)	0.0 (−1.1 to 1.1)

Abbreviations: DID, difference in differences; RHEA, Reproductive Health Equity Act.

^a^ Models were adjusted for age, metropolitan status, and federal poverty level category. The SEs were clustered at the clinic level. The parallel trends assumption was met.

^b^ Highly effective methods included intrauterine device or intrauterine system and implant.

^c^ Moderately effective methods included injectable contraceptive, oral contraceptive pills, contraceptive patch, and contraceptive ring.

Results of a sensitivity analysis excluding visits from quarter 1 of 2020 were similar to those of the primary analysis; we estimated a significant 2.1 percentage point (95% CI, 0.3-3.9 percentage points) increase in prevalence of moderately and highly effective method use for Latina patient visits compared with non-Latina patient visits. There were no significant differences noted for the other outcomes of interest (eTable in Supplement 1).

## Discussion

Our analysis showed that comprehensive contraception coverage with no patient cost sharing was associated with a slightly decreased disparity in prevalence of moderately or highly effective contraceptive methods, with an adjusted increased prevalence of 1.9 percentage points for Latina women compared with non-Latina women in Oregon when DID methods were used. However, results did not show any differences in change in prevalence of highly effective contraceptive methods for Latina women compared with non-Latina women or adoption and continuation of highly or moderately and highly effective methods. Prior studies using National Survey of Family Growth data before and after implementation of the ACA showed overall increased use of highly effective contraceptive methods but no association with ethnicity^29^ or improvement in moderately and highly effective contraceptive use for Latina women compared with White women.^30^

While cost is associated with contraceptive use,^12,13^ it is not the only factor affecting contraceptive choice. There is also evidence that race-based discrimination within the health system perpetuates health disparities, including nonuse of contraception.^31^ Higher rates of intimate partner violence or reproductive coercion,^10,32^ preferences for method features,^11^ and perceptions of being unable to plan a pregnancy^33^ may lead some women to choose a non–highly effective method. Conversely, US clinicians and contraceptive programs have engaged in coercive practices promoting or mandating use of highly effective contraceptive methods for patients with low income that may undermine patient autonomy and increase mistrust in the health care system.^34,35^ For these reasons, we chose prevalence of moderately and highly effective method use as opposed to prevalence of highly effective method use alone as our primary outcome. Notably, in Oregon, we found that Latina women had a higher prevalence of highly effective method use than non-Latina women, although we do not have information on patient preference.

Despite this finding, expanded contraceptive coverage since ACA implementation led to improved access to family planning services^36^ and increased utilization of highly effective contraceptive methods for both Latina and non-Latina patients.^15^ However, gaps in coverage remain for populations such as undocumented immigrants, who disproportionately rely on publicly funded clinics for contraceptive access.^17,37^ Title X, unlike Medicaid, does not require proof of US citizenship or an eligible immigration status for coverage. The Oregon withdrawal from Title X may have had disproportionate ramifications for receipt of contraceptive care for Latina patients, as Title X funds covered approximately a quarter of contraceptive visits for Latina patients compared with merely 5% of visits for non-Latina patients. However, state funds using a more responsive clinic fee-for-service reimbursement model appeared to successfully supplant federal public family planning Title X funds and enable continuation of high-quality family planning services for all individuals with low income despite Oregon’s withdrawal in 2019.

Our study thus adds to the literature showing that expanding reproductive health coverage for individuals not eligible for Medicaid coverage is associated with improved contraceptive uptake.^23,38^ Similar state-funded comprehensive family planning programs have additionally proven to be cost-effective,^39^ which may ensure reliable contraceptive coverage for individuals who do not meet criteria for Medicaid and decreased reliance on federal funds that may be subject to instability with changing administrations.

### Limitations

Our study has several important limitations. We did not have data for several key factors associated with contraceptive use, including parity, comprehensive data on pregnancy intentions, or desired contraceptive method^5,11^; thus, we could not adjust for these variables. We could not assess patient satisfaction with care or their reproductive autonomy through a measure such as the Person-Centered Contraceptive Counseling scale,^40,41^ although clinicians within the Oregon RH Program are encouraged to use a shared decision-making approach with patients and provide them with the method of their choosing. We were also unable to use immigration status as our primary independent variable, although RHEA legislation primarily benefits individuals who are unable to demonstrate proof of US citizenship or eligible immigration status. This inability was due to inconsistent data collection for immigration status prior to RHEA implementation.

Our data are also from a single state, which affects generalizability. Oregon has invested for decades in a strong network of publicly funded family planning clinics, in Title X, and in Medicaid expansion. The proportion of clinic visits at which a patient adopted or continued a moderately and highly effective contraceptive method was greater than 75% for patients in Oregon’s RH Program both prior to and after RHEA implementation, which already exceeds the target objective of 69.3% set in Healthy People 2020.^42^ Implementation of similar policies in other states may show even further reductions in disparities in moderately and highly effective contraception.

## Conclusions

In this historical cohort study, ensuring no-cost comprehensive coverage using a fee-for-service funding model for all low-income Oregonians regardless of immigration status was associated with increased prevalence of use of moderately and highly effective contraception among Latina individuals compared with non-Latina individuals, thereby mitigating ethnic disparities in contraception use. Ensuring contraceptive coverage for all individuals through similar state policies or robust and well-funded federal Title X programs may help individuals access the contraceptive methods and services they prefer. Such policies have been shown to be cost-effective,^6^ may increase reproductive autonomy, and may help reduce intergenerational disparities in health outcomes.^43,44^

## Notes

Supplement 1. **eFigure 1.** Cohort Derivation for Contraceptive Visits for Women Ages 12-51 at Risk of Pregnancy in Oregon Reproductive Health Program, 2016-2020 **eFigure 2.** Unadjusted Trends for Latina Compared to Non-Latina Patients by Quarter 2016-2020, and P-Values for Pre-test of Parallel Trends **eTable.** Sensitivity Analysis Excluding Quarter 1 of 2020: Changes in Contraceptive Outcomes for Latina Women Compared to Non-Latina Women in Oregon Following RHEA (2016-2019), n = 282 171

Supplement 2.
Data Sharing Statement
