# Supplementary material for: Association of State Funding for Comprehensive Reproductive Health Care With Use of Contraception Among Latina Patients and Non-Latina Patients in Oregon
Source: JAMA Health Forum. 2023 Jul 28;4(7):e232144. doi: 10.1001/jamahealthforum.2023.2144 (PMC10383011; doi:10.1001/jamahealthforum.2023.2144)
Supplement: Supplement 1. — eFigure 1. Cohort Derivation for Contraceptive Visits for Women Ages 12-51 at Risk of Pregnancy in Oregon Reproductive Health Program, 2016-2020 eFigure 2. Unadjusted Trends for Latina Compared to Non-Latina Patients by Quarter 2016-2020, and P-Values for Pre-test of Parallel Trends eTable. Sensitivity Analysis Excluding Quarter 1 of 2020: Changes in Contraceptive Outcomes for Latina Women Compared to Non-Latina Women in Oregon Following RHEA (2016-2019), n = 282 171 [file jamahealthforum-e232144-s001.pdf]

Supplementary Online Content

Cohen MA, Boniface ER, Skye M, Linz R, Pedhiwala N, Rodriguez MI. Association of state funding for comprehensive reproductive health care with use of contraception among Latina patients and non-Latina patients in Oregon. *JAMA Health Forum*. 2023;4(7):e232144. doi:10.1001/jamahealthforum.2023.2144

eFigure 1. Cohort Derivation for Contraceptive Visits for Women Ages 12-51 at Risk of Pregnancy in Oregon Reproductive Health Program, 2016-2020

eFigure 2. Unadjusted Trends for Latina Compared to Non-Latina Patients by Quarter 2016-2020, and P-Values for Pre-test of Parallel Trends

eTable. Sensitivity Analysis Excluding Quarter 1 of 2020: Changes in Contraceptive Outcomes for Latina Women Compared to Non-Latina Women in Oregon Following RHEA (2016-2019), n=282 171

This supplementary material has been provided by the authors to give readers additional information about their work.

© 2023 Cohen MA et al. *JAMA Health Forum.*

eFigure 1. Cohort derivation for contraceptive visits for women ages 12-51 at risk of pregnancy in Oregon Reproductive Health Program, 2016-2020.

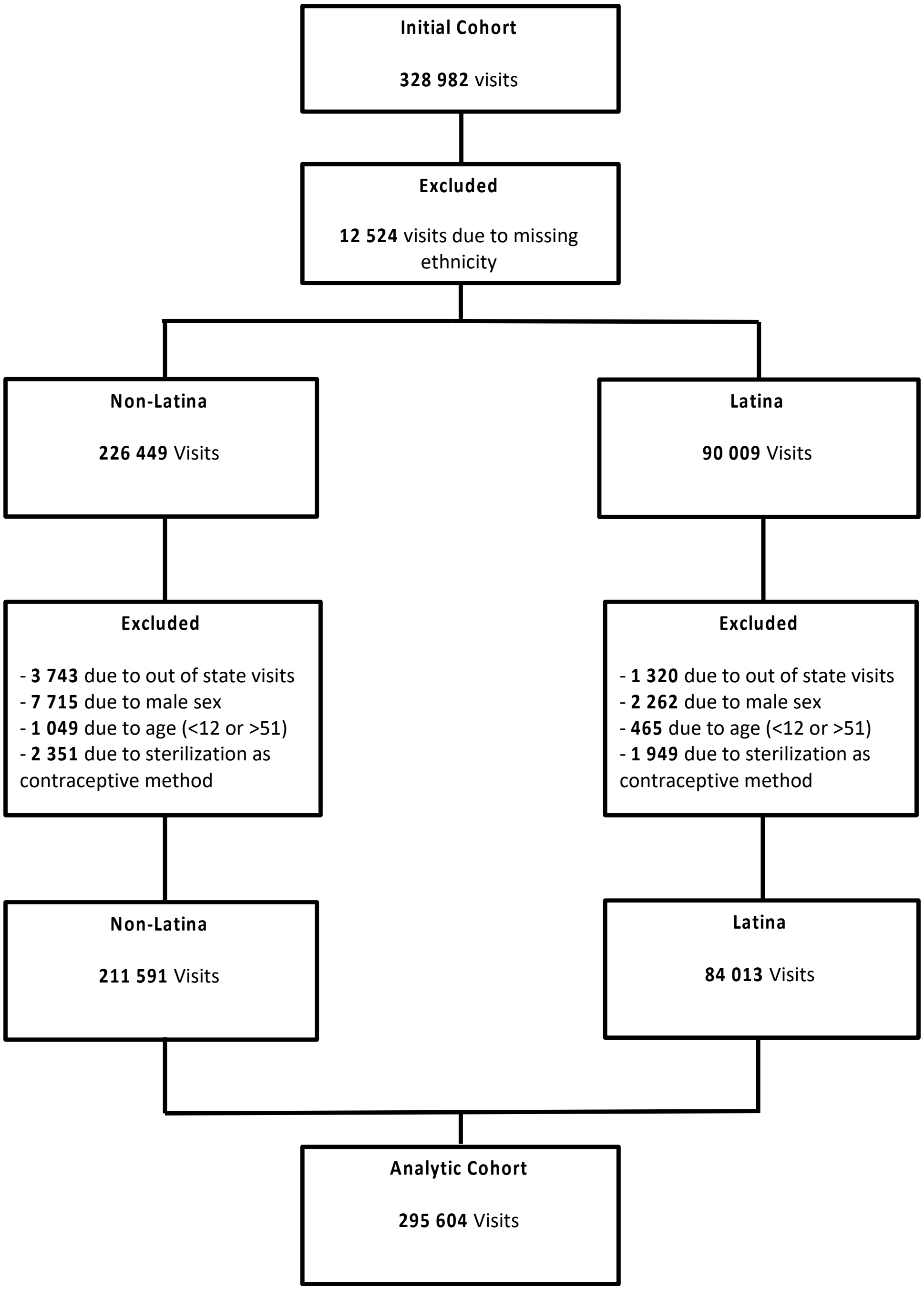

© 2023 Cohen MA et al. *JAMA Health Forum.*

eFigure 2. Unadjusted trends for Latina compared to non-Latina patients by quarter 2016-2020, and p-values for pre-test of parallel trends

Panel a) Prevalence of moderate and highly effective method, p = 0.292; b) Adoption or continuation of moderate and highly effective method, p = 0.312; c) Prevalence of highly effective method, p = 0.427; and d) Adoption or continuation of highly effective method, p = 0.156.

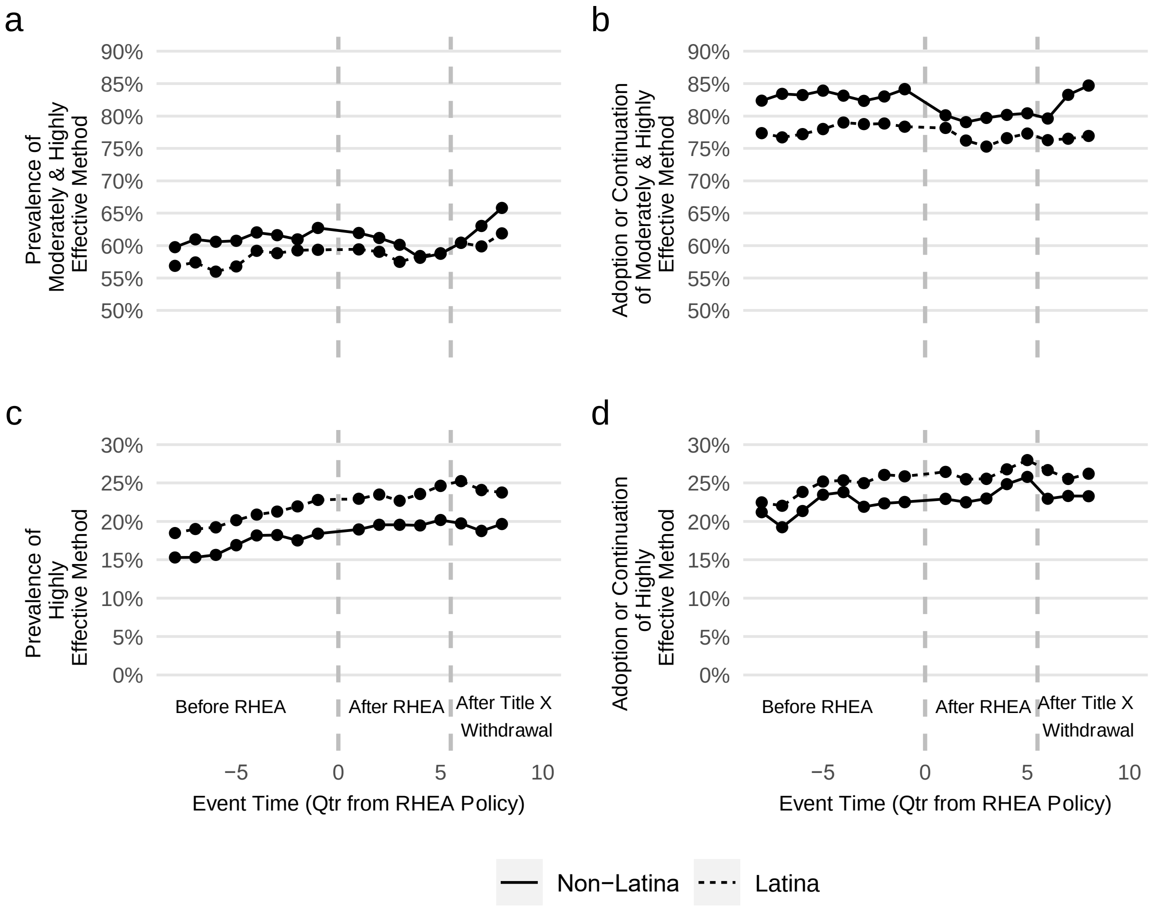

© 2023 Cohen MA et al. *JAMA Health Forum.*

eTable. Sensitivity analysis excluding quarter 1 of 2020: Changes in contraceptive outcomes for Latina women compared to non-Latina women in Oregon following RHEA (2016-2019), n=282 171.

Outcome	Pre-Policy Proportion among non-Latina women % (N=111 924)	Post-Policy Proportion among non-Latina women % (N=90 912)	Pre-Policy Proportion among Latina women (N=40 808)	Post-Policy Proportion among Latina women (N=38 527)	Adjusted Difference-in-Difference Estimate Percentage Points[c] (95% CI)
Moderately and highly effective method, prevalence[a,b]	68 413 (61.1)	54 808 (60.3)	23 646 (57.9)	22 754 (59.1)	2.1 (0.3 - 3.9)
Moderately and highly effective method, adoption and continuation[a,b]	93 101 (83.2)	72 962 (80.3)	31 840 (78.0)	29 529 (76.6)	1.4 (-0.3 – 3.1)
Highly effective method, prevalence[a]	18 875 (16.9)	17 727 (19.5)	8 343 (20.4)	9 187 (23.8)	0.7 (-0.3 - 1.7)
Highly effective method, adoption and continuation[a]	24 600 (22.0)	21 607 (23.8)	9 983 (24.5)	10 184 (26.4)	0.0 (-1.1 - 1.1)

[a] Highly effective methods include: IUD/IUS, and implant

[b] Moderately effective methods include: Injectable, oral contraceptive pills, contraceptive patch, and contraceptive ring

[c] Models were adjusted for age, metropolitan status, and federal poverty level category. Standard errors clustered at clinic level. Parallel trends assumption met.

© 2023 Cohen MA et al. *JAMA Health Forum.*
