# Supplementary material for: Association of State Funding for Comprehensive Reproductive Health Care With Use of Contraception Among Latina Patients and Non-Latina Patients in Oregon
Source: JAMA Health Forum. 2023 Jul 28;4(7):e232144. doi: 10.1001/jamahealthforum.2023.2144 (PMC10383011; doi:10.1001/jamahealthforum.2023.2144)
Supplement: Supplement 2. — Data Sharing Statement [file jamahealthforum-e232144-s002.pdf]

Data Sharing Statement

Cohen. Association of State Funding for Comprehensive Reproductive Health Care With Use of Contraception Among Latina Patients and Non-Latina Patients in Oregon. *JAMA Health Forum*. Published July 28, 2023. doi:10.1001/jamahealthforum.2023.2144

Data
Data available: No

Additional Information
Explanation for why data not available: Access to the data requires a data use agreement with the Oregon Health Authority
